# Comparing pedigree and genomic inbreeding coefficients, and inbreeding depression of reproductive traits in Japanese Black cattle

**DOI:** 10.1186/s12864-023-09480-5

**Published:** 2023-07-05

**Authors:** Motohide Nishio, Keiichi Inoue, Shinichiro Ogawa, Kasumi Ichinoseki, Aisaku Arakawa, Yo Fukuzawa, Toshihiro Okamura, Eiji Kobayashi, Masaaki Taniguchi, Mika Oe, Kazuo Ishii

**Affiliations:** 1 Institute of Livestock and Grassland Science, NARO, Tsukuba, Ibaraki, 3050901 Japan; 2 University of Miyazaki, Miyazaki, Miyazaki 889-2192 Japan; 3 National Livestock Breeding Center, Nishigo, Fukushima 961-8511 Japan

**Keywords:** Inbreeding depression, Pedigree inbreeding, Genomic inbreeding, Chromosomal inbreeding

## Abstract

**Background:**

Pedigree-based inbreeding coefficients have been generally included in statistical models for genetic evaluation of Japanese Black cattle. The use of genomic data is expected to provide precise assessment of inbreeding level and depression. Recently, many measures have been used for genome-based inbreeding coefficients; however, with no consensus on which is the most appropriate. Therefore, we compared the pedigree- (${F}_{PED}$) and multiple genome-based inbreeding coefficients, which were calculated from the genomic relationship matrix with observed allele frequencies (${F}_{GRM}$), correlation between uniting gametes (${F}_{UNI}$), the observed vs expected number of homozygous genotypes (${F}_{HOM}$), runs of homozygosity (ROH) segments (${F}_{ROH}$) and heterozygosity by descent segments (${F}_{HBD}$). We quantified inbreeding depression from estimating regression coefficients of inbreeding coefficients on three reproductive traits: age at first calving (AFC), calving difficulty (CD) and gestation length (GL) in Japanese Black cattle.

**Results:**

The highest correlations with ${F}_{PED}$ were for ${F}_{ROH}$ (0.86) and ${F}_{HBD}$ (0.85) whereas ${F}_{GRM}$ and ${F}_{UNI}$ provided weak correlations with ${F}_{PED}$, with range 0.33–0.55. Except for ${F}_{GRM}$ and ${F}_{UNI}$, there were strong correlations among genome-based inbreeding coefficients ($\ge$ 0.94). The estimates of regression coefficients of inbreeding depression for ${F}_{PED}$ was 2.1 for AFC, 0.63 for CD and -1.21 for GL, respectively, but ${F}_{PED}$ had no significant effects on all traits. Genome-based inbreeding coefficients provided larger effects on all reproductive traits than ${F}_{PED}$. In particular, for CD, all estimated regression coefficients for genome-based inbreeding coefficients were significant, and for GL, that for ${F}_{UNI}$ had a significant.. Although there were no significant effects when using overall genome-level inbreeding coefficients for AFC and GL, ${F}_{ROH}$ provided significant effects at chromosomal level in four chromosomes for AFC, three chromosomes for CD, and two chromosomes for GL. In addition, similar results were obtained for ${F}_{HBD}$.

**Conclusions:**

Genome-based inbreeding coefficients can capture more phenotypic variation than ${F}_{PED}$. In particular, ${F}_{ROH}$ and ${F}_{HBD}$ can be considered good estimators for quantifying inbreeding level and identifying inbreeding depression at the chromosome level. These findings might improve the quantification of inbreeding and breeding programs using genome-based inbreeding coefficients.

**Supplementary Information:**

The online version contains supplementary material available at 10.1186/s12864-023-09480-5.

## Background

Over recent decades, Japanese Black cattle populations have experienced greatly improved meat quality due to abundant marbling caused by intramuscular fat deposits. This was accomplished by the intensive use of a few excellent sires with high estimated breeding values for marbling score. Such a rapid improvement resulted in decreasing the effective population size and increasing the amount of inbreeding. Nomura et al. [1] revealed that the effective population size decreased sharply to 17.2, and average inbreeding coefficients increased to 5.4% during 1985–1997 in Japanese Black cattle using the pedigree files of more than 1,800,000 animals. Recently, genomic prediction has been applied to Japanese Black cattle [2, 3]. Genomic prediction can reduce the generational rates of inbreeding by accounting for Mendelian sampling with single nucleotide polymorphism (SNP) information [4], but reduces the generation intervals due to accurately predicting breeding values at birth [5], which would result in increasing inbreeding per year. In fact, yearly inbreeding has increased in Dutch-Flemish [6] and North American populations [7] in dairy cattle. In the future, genomic prediction will accelerate the accumulation of inbreeding in the Japanese Black cattle population. Increased inbreeding often has detrimental effects on the performance and fitness of progeny [8, 9], in a phenomenon known as “inbreeding depression,” which is caused by the accumulation of deleterious mutations [10]. The precise assessment of inbreeding is critical in the design of a breeding program to control the increase in inbreeding levels and thereby control inbreeding depression.

The inbreeding coefficient is a criterion for the management of populations and for the study of inbreeding depression, and is defined as the probability that two alleles in an individual are identical by descent (IBD) relative to a base population where all alleles are assumed unrelated [11]. The inbreeding coefficient is usually calculated from the pedigree, and the probabilities that a pair of alleles is IBD is estimated from statistical expectations [12]. Estimation of the pedigree-based inbreeding coefficient depends on the depth and reliability of the pedigree. More recently, increasing availability of genomic information, particularly SNP data, has provided the opportunity to assess inbreeding even when no pedigree is available. The probability of an allele at a locus being IBD can be estimated by direct inference from the alleles inherited by an individual, which can be performed for tens of thousands or more SNPs covering the whole genome. Thus, use of genomic data is expected to provide a precise assessment of inbreeding. Nowadays, several genome-based inbreeding coefficients have been proposed and can be broadly classified into three types of approaches: by a SNP-by-SNP evaluation of the level of homozygosity [13], by examining identical by state that summarizes SNP-by-SNP information using a genomic relationship matrix [14, 15] and by using segment-based homozygosity [16]. The above SNP-based measures detected inbreeding depression more effectively than pedigree-based estimates in a simulation study [17] and a meta-analysis of different studies [18]. In particular, the inbreeding coefficient based on runs of homozygosity (ROH) was recently reported to be more accurate for assessing individual inbreeding levels than other inbreeding coefficient estimators [19, 20]. However, there is no consensus on the most appropriate approach [21].

An inbreeding effect has generally been included in statistical models for genetic evaluation of Japanese Black cattle. Several studies reported a pedigree-based inbreeding coefficient associated with economic traits in Japanese Black cattle. For example, Uchida et al. [22] reported that linear regression coefficients for calves’ growth traits against the inbreeding coefficients of their dams were significant and negative in regard to birth weight and market weight per day. Oyama et al. [23] and Ogawa and Satoh [24] reported that a high inbreeding coefficient of cows contributed to the extension of the calving interval. Atagi et al. [25] detected detrimental effects of inbreeding in semen production traits. Nishi et al. [26] observed a positive linear relationship between the defect incidence and inbreeding coefficients in muscle steatosis, bruising and trim loss, which all lower carcass value. However, few studies have investigated inbreeding depression using a genome-based inbreeding coefficient in Japanese Black cattle. For genomic prediction, the heterozygosity rate of SNP genotypes was included as a covariate in semen production traits [27] and carcass traits [28], but they considered other indices describing degree of inbreeding. Although Suezawa et al. [29] used an inbreeding coefficient based on ROH for evaluating genetic diversity in Japanese Black cows in the islands of Okinawa Prefecture, the effects of ROH-based inbreeding coefficients using actual records were not investigated.

Recently, reproductive traits in Japanese Black cattle have been of increasing interest because they affect the profitability of beef production systems over a long period. Ogawa et al. [30] suggested that earlier age at first calving (AFC) would increase the lifetime profit of Japanese Black cows through producing more feeder cattle. Stillbirth and dystocia also have a substantial impact on economic losses due to increasing labor and veterinary costs and loss of production and impaired reproductive performance of cows. Dystocia accounted for an increasing annual percentage of sickness and injury incidents in the beef industry, by 0.5 percentage points from 2008 (2.8%) to 2018 (3.3%) in Japanese Black cattle [31]. Reproductive traits are usually more affected by inbreeding depression than other traits.

This study compares several methods for estimating the inbreeding coefficient based on pedigree and SNP information and investigates a suitable method for estimating inbreeding depression on reproductive traits in Japanese Black cattle.

## Material and methods

### Animals, phenotypes and genotypes

Animal Care and Use Committee approval was not needed for this study because the data were acquired from an existing database of the National Livestock Breeding Center (NLBC), Japan.

This study comprised 2,583 Japanese Black cows with phenotypic records of reproductive traits including AFC, calving difficulty (CD) and gestation length (GL) from the four breeding stations of the NLBC. The CD was scored on a 1–5 scale by NLBC technicians: 1 = no problem or unobserved, 2 = slight problem, 3 = cow needed assistance, 4 = considerable force used to deliver calf and 5 = extremely difficult birth. For CD, fifth category was removed in our analysis. Also, we removed records exceeded 3 standard deviations for AFC. Phenotyped cows were born between 1998 and 2020 and genotyped using GeneSeek Genomic Profiler: GGP BovineLD v4.0, which had 30,105 SNPs (Illumina, San Diego, CA, USA). These genotypes were imputed to BovineSNP50 BeadChip BeadsChip (Illumina) using Beagle v4.0 software [32]. The reference population for imputation comprised the BovineSNP50 BeadChip genotypes of 651 Japanese Black cattle. The detail of the reference population is described in Watanabe [33] and Ogawa et al. [34]. The quality of imputation using this reference population was valid for genomic prediction and genome-wide association study [35, 36]. Moreover, several studies previously evaluated the genetic diversity and structure in Japanese Black cattle using BovineSNP50 BeadChip or GGP BovineLD v4.0 [37–39]. All SNP were filtered for call rate < 95%, minor allele frequency (MAF) < 0.01 and extreme deviation from Hardy–Weinberg equilibrium (*p* < 0.0001). After imputation and quality control, there were 2,535 genotyped animals and 34,481 SNP markers available in the final dataset. Phenotypic averages ± SDs were 790 ± 98 days, 1.40 ± 0.76 and 285.6 ± 4.7 days for AFC, CD and GL, respectively.

### Estimation of inbreeding coefficients

A pedigree file was constructed by tracing back up to seventeenth generations of ancestors and included 16,406 individuals. The pedigree-based inbreeding coefficient (${F}_{PED}$) was calculated with the algorithm of Meuwissen and Luo [40] using our own program coded by Fortran. In addition, we calculated effective.

We used seven different estimators of inbreeding coefficients based on genomic information: ${F}_{GRM}$, ${F}_{UNI}$, ${F}_{HOM}$, ${F}_{ROH}$, ${F}_{ROH\_30}$, ${F}_{ROH\_15}$ and ${F}_{HBD}$.

The first estimator ${F}_{GRM}$ was calculated from diagonal elements of the genomic relationship matrix (GRM). The form of ${F}_{GRM}$ follows:

$${F}_{GRM}=diag\left(\mathbf{G}\right)-1,$$

where $\mathbf{G}$ is the GRM built according to VanRaden’s first method [14]. The GRM can be calculated from the following:

$$\mathbf{G}=\frac{\mathbf{M}{\mathbf{M}}^{\mathbf{^{\prime}}}}{\sum_{j=1}^{m}2{p}_{j}(1-{p}_{j})},$$

where $\mathbf{M}=\mathbf{X}-2{p}_{j}$, $\mathbf{X}$ is the $n\times m$ matrix of the genotypes coded by the number of the second allele, $n$ is the number of genotyped animals, $m$ is the number of markers and ${p}_{j}$ is the frequency of the second allele at locus $j$. The GRM method is appropriate when the allele frequencies used are those in the founder population. To mimicking the founder population, we used the allele frequencies in the animals genotyped in the first four years from 2001 to 2004 because the generation interval of cow in this population was 3.98. In this procedure, we did not use animals born from 1998 to 2000 because its number was too small. The ${F}_{GRM}$ was calculated by our own program coded in the R language.

The ${F}_{UNI}$ estimate was calculated from the correlation between uniting gametes following Yang et al. [15]:

$${F}_{UNI}=\frac{1}{m}\sum_{j=1}^{m}\frac{{\mathbf{X}}_{j}^{2}-\left(1+2{p}_{j}\right){\mathbf{X}}_{j}+2{p}_{j}^{2}}{2{p}_{j}(1-{p}_{j})}.$$

The ${F}_{HOM}$ estimate was based on the observed vs expected number of homozygous genotypes and was calculated following Wright [41]:

$${F}_{HOM}=1-\frac{1}{m}\sum_{j=1}^{m}\frac{{\mathbf{X}}_{j}\left(2-{\mathbf{X}}_{j}\right)}{2{p}_{j}(1-{p}_{j})}$$

The ${F}_{UNI}$ and ${F}_{HOM}$ were calculated with the same allele frequencies as ${F}_{GRM}$. We estimated ${F}_{UNI}$ and ${F}_{HOM}$ using our own program coded in the R language.

The ROH are defined as continuous and uninterrupted chromosome portions showing homozygosity at all loci [16]. The inbreeding coefficient based on ROH was defined as the total length of ROH divided by the overall length of the autosomal genome covered by SNPs. We calculated ${F}_{ROH}$ using the sliding window method for detection of ROH segments. In the sliding window approach, the following parameters and thresholds were applied to reduce the number of spurious ROH detected: (i) the minimum number of consecutive homozygous SNP included in the ROH ($L$) was 60, (ii) the minimum region length that constituted the ROH was 1 Mbp, (iii) the minimum density of SNP in a genome window was 1 SNP every 100 kbp, (iv) the maximum allowed distance between consecutive SNPs was 1 Mbp, (v) the number of heterozygous SNPs that were allowed in the ROH was 1, (vi) scanning window size was 15 SNPs and (vii) scanning window threshold was 0.05. In this study, the value of $L$ was determined following the formula proposed by Lencz et al. [42] and adapted by Purfield et al. [43]:

$$\frac{{log}_{e}\frac{\alpha }{{n}_{s}{n}_{i}}}{{log}_{e}\left(1-het\right)},$$

where $\alpha$ is the percentage of false-positive ROH, and was set at 0.05; ${n}_{s}$ is the number of genotyped SNPs per individual; ${n}_{i}$ is the number of genotyped individuals; and $het$ is the mean heterozygosity across all SNPs. The stringent criterion of $L$ reduces false-positive ROH caused by linkage disequilibrium, but also reduces the detection of short ROH segments, which contain deleterious alleles. Discarding such short ROH segments results in underestimation of the ROH originating from more distant ancestors and might lead to substantial bias in estimation of inbreeding depression. Thus, we added two inbreeding coefficients ${F}_{ROH\_30}$ and ${F}_{ROH\_15}$ in which the values of $L$ were set to 30 and 15, respectively. For all ROH-based inbreeding coefficients, no pruning was performed based on MAF and linkage disequilibrium to avoid biases introduced by the practice [44]. The ROH-based inbreeding coefficients were calculated using the R package detectRUNS [45].

Heterozygosity by descent (HBD) or autozygosity was defined as an IBD homozygosity at the DNA level. Generally, the history of a population is complex, and common ancestors belong to different generations. This frequently occurs in small populations, or in populations under strong selection. In this connection, Druet and Gautier [46] presented an approach to solving this problem based on the HBD multiple class model. Unlike ROH, the sequence of HBD and non-HBD segments is modeled using the hidden Markov model. As a result, total autozygosity can be divided according to the age of the inbreeding event. The probability of staying in a particular state is calculated as ${e}^{-{R}_{k}}$, where ${R}_{k}$ is the rate specific to the $k$ th class. This means that the length of an HBD segment of any class is exponentially distributed with rate ${R}_{k}$. The ${F}_{HBD}$ estimate was calculated from the proportion of the genome in HBD segments and obtained using the R package RZooROH [47]. In this study, we determined the model with 10 HBD classes following predefined default rates in the RZooROH package.

Pairwise correlations ($\rho$) between the different measurements of inbreeding were computed to assess their relatedness. To investigate the similarity between pedigree- and genome-based inbreeding coefficients, we implemented the regression of ${F}_{PED}$ on genome-based inbreeding coefficients. Moreover, principal component analysis (PCA) was performed on all inbreeding coefficients using the R package prcomp.

### Inbreeding depression analysis

Inbreeding depression was estimated separately for each reproductive trait using the following linear mixed model:

$$\mathbf{y}=\mathbf{X}\mathbf{b}+\beta \mathbf{F}+\mathbf{Z}\mathbf{u}+\mathbf{e}$$

where $\mathbf{y}$ is the vector of observed phenotypes; $\mathbf{b}$ is the vector of fixed effects including the sex of calves (two levels: male and female),month of calving (12 levels) and the vector of contemporary group effects which includes herd-year at calving (68 levels) for all traits, and linear and quadratic covariates of AFC for CD and GL; $\beta$ is the coefficient of the linear regression on $\mathbf{F}$; $\mathbf{F}$ is the vector of inbreeding coefficients from pedigree and genomic data; $\mathbf{u}$ is the vector of random genetic additive effects; $\mathbf{e}$ is the vector of random residuals; and $\mathbf{X}$ and $\mathbf{Z}$ are the known incident matrices relating fixed and random effects to observations. The variance of the random effects was assumed to be $\mathbf{u}\sim N(0, \mathbf{A}{\sigma }_{u}^{2})$ for ${F}_{PED}$ or $\mathbf{u}\sim N(0, \mathbf{G}{\sigma }_{u}^{2})$ for genome-based inbreeding coefficients and $\mathbf{e}\sim N(0,\mathbf{I}{\sigma }_{e}^{2})$, where ${\sigma }_{u}^{2}$ is the additive genetic variance, ${\sigma }_{e}^{2}$ is the residual variance, $\mathbf{A}$ is the numerator relationship matrix, $\mathbf{G}$ is the GRM built with the same SNP panel used to compute the measure of inbreeding being tested and $\mathbf{I}$ is an identity matrix of dimension of equal to the number of observations.

Unlike a pedigree-based inbreeding coefficient, genome-based inbreeding coefficients can be partitioned into the relative contribution of each autosomal chromosome. To investigate the effect of chromosome-specific inbreeding depression, we computed chromosomal inbreeding coefficients of ${F}_{ROH}$ and ${F}_{HBD}$ from the ratios of chromosome lengths covered by ROH and HBD to the overall chromosome length. The model presented above was modified by replacing the genome-wide inbreeding coefficient by the chromosomal inbreeding coefficient:

$$\mathbf{y}=\mathbf{X}\mathbf{b}+\sum_{i=1}^{29}{\beta }_{i}{{\varvec{F}}}_{{\varvec{i}}}+\mathbf{Z}\mathbf{u}+\mathbf{e}$$

where ${\beta }_{i}$ is the coefficient of the linear regression on ${\mathbf{F}}_{{\varvec{i}}}$, and ${\mathbf{F}}_{{\varvec{i}}}$ is the vector of inbreeding coefficient of $i$ th chromosome.

Single-trait analysis was carried out using the BGLR package [48] in the R language, as a member of reproducing kernel Hilbert space regression models. The Markov chain Monte Carlo (MCMC) was run for 100,000 cycles with a 50,000 burn-in period and a thinning interval of 10. Convergence of the MCMC chain was confirmed in the coda package [49] in the R language. Regression coefficients and corresponding posterior standard deviations (PSDs) for inbreeding coefficients were obtained from output. The effect of inbreeding on reproductive traits was assessed based on the significance of its associated regression coefficients ($\widehat{\beta }$) using the t-statistic unit ($\widehat{\beta }/PSD$).

## Results

### Patterns of ROH segments

**Fig. 1:**
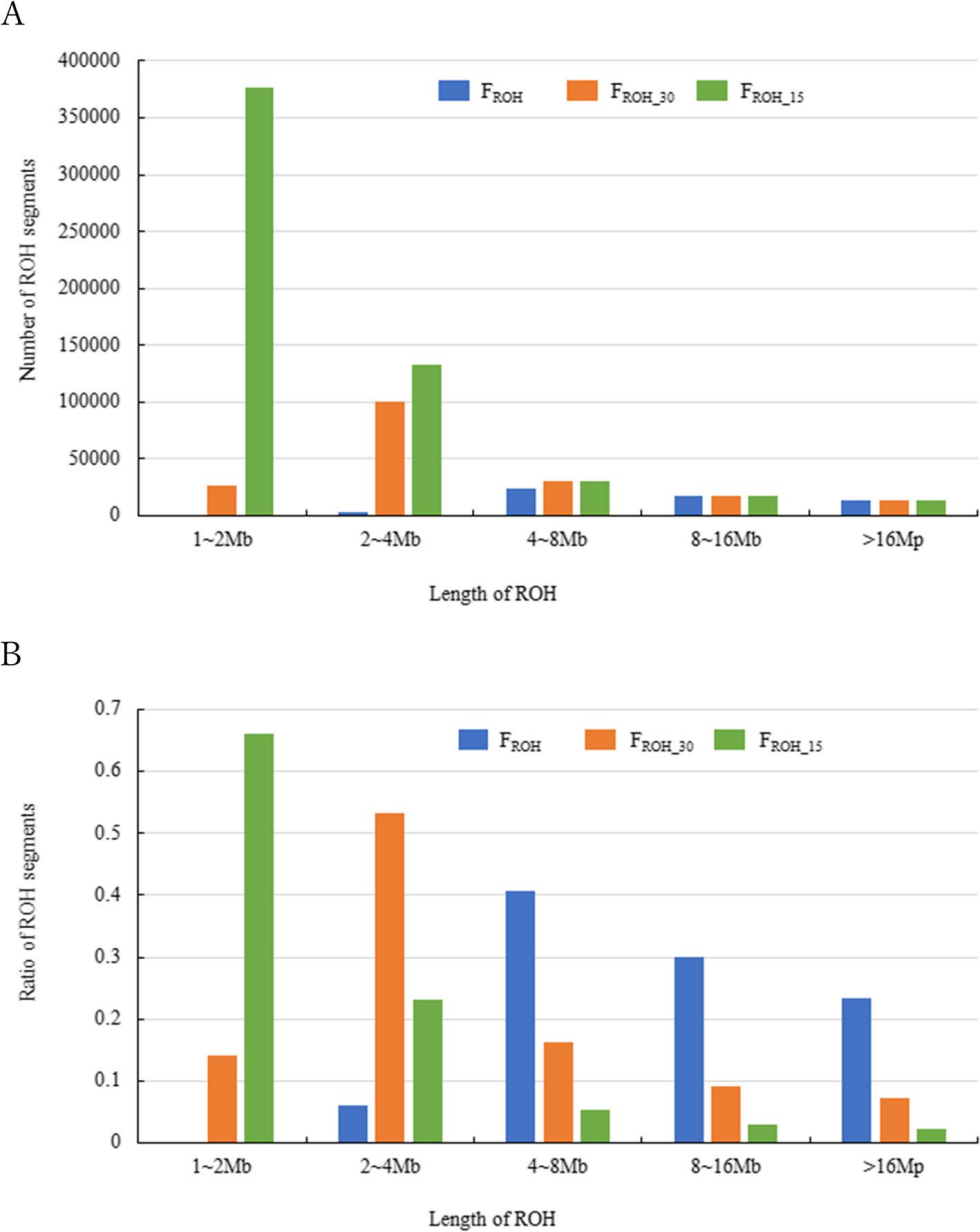
Distributions of the number of ROH segments (A) and ratio of ROH segments (B) using different minimum number of consecutive homozygous SNPs included in the ROH: 60 (${F}_{ROH}$), 30 (${F}_{ROH\_30}$) and 15 (${F}_{ROH\_15}$)

We used three parameter settings for the value of $L$ for ROH-based inbreeding coefficients (${F}_{ROH}$, ${F}_{ROH\_30}$ and ${F}_{ROH\_15}$). The total numbers of ROH segments for ${F}_{ROH}$, ${F}_{ROH\_30}$ and ${F}_{ROH\_15}$ were 57,892, 188,092 and 569,980, respectively. The distributions of the number and ratio of ROH segments are described in Fig. 1. The numbers of ROH segments longer than 8 kb for all ROH-based inbreeding coefficients were the same whereas numbers of ROH segments shorter than 4 kb greatly increased with the reduction of $L$. This resulted in the ratios of short ROH segments for ${F}_{ROH\_30}$ and ${F}_{ROH\_15}$ being high compared to ${F}_{ROH}$.

### Comparison of inbreeding coefficients

**Fig. 2:**
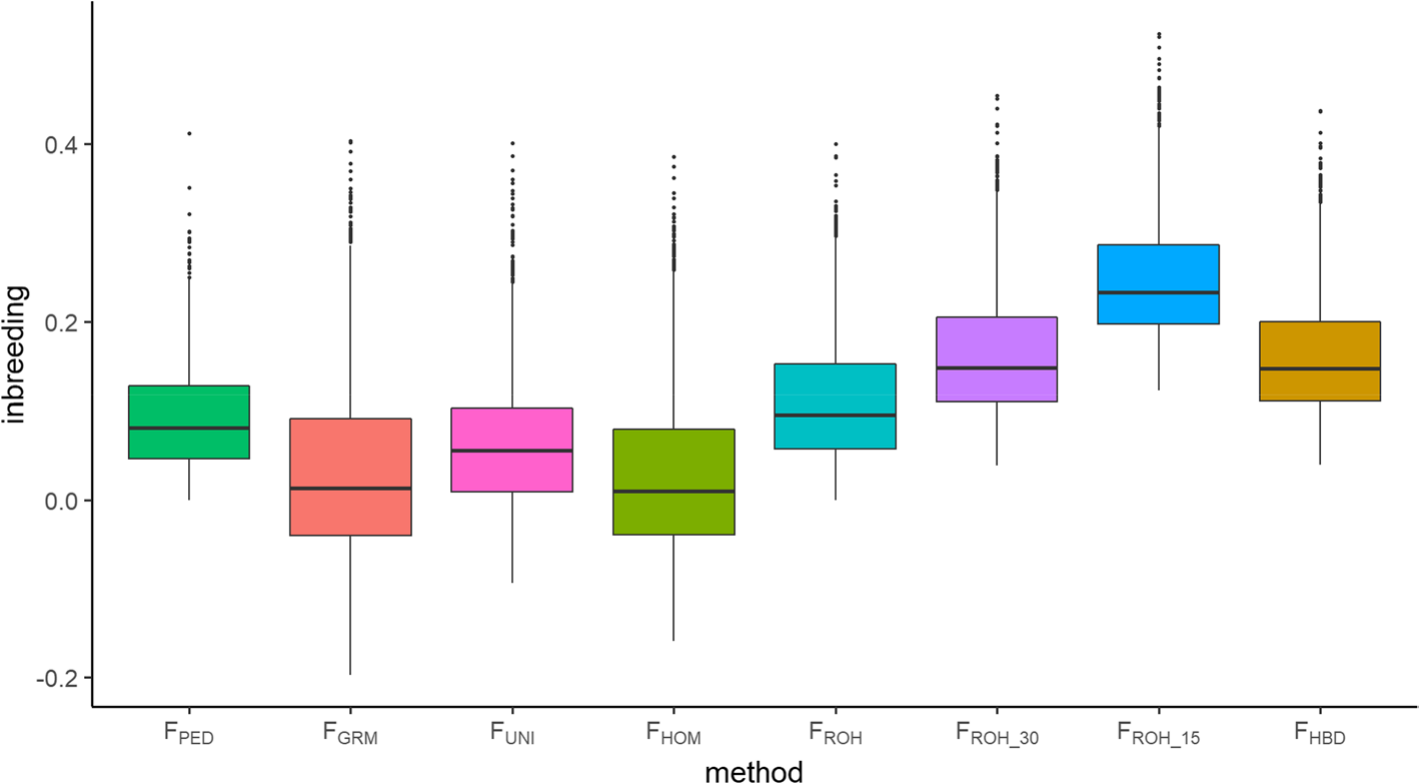
Box plots of different inbreeding coefficients. ${F}_{PED}$, pedigree-based inbreeding coefficient; ${F}_{GRM}$, inbreeding coefficient based on genomic relationship matrix; ${F}_{UNI}$, inbreeding coefficient based on correlation between uniting gametes; ${F}_{HOM}$, inbreeding coefficient based on the observed vs expected number of homozygous genotypes; ${F}_{ROH}$, inbreeding coefficient based on ROH; ${F}_{ROH\_30}$, inbreeding coefficient based on ROH when minimum number of consecutive homozygous SNP included in the ROH was 30; ${F}_{ROH\_15}$ inbreeding coefficient based on ROH when minimum number of consecutive homozygous SNP included in the ROH was 15; ${F}_{HBD}$, inbreeding coefficient based on HBD

The statistics of all inbreeding coefficients are summarized in Table 1 and Fig. 2. The range of classical ${F}_{PED}$ was 0.000–0.412 with mean of 0.093. The minimum, mean and median for ${F}_{GRM}$, ${F}_{UNI}$ and ${F}_{HOM}$ were smaller than those for ${F}_{PED}$. The ranges for ROH-based inbreeding coefficients were 0.000–0.400, 0.039–0.454 and 0.122–0.524 for ${F}_{ROH}$, ${F}_{ROH\_30}$ and ${F}_{ROH\_15}$, respectively. Small values of $L$ resulted in high inbreeding coefficients but the SDs were almost the same for ${F}_{ROH}$, ${F}_{ROH\_30}$ and ${F}_{ROH\_15}$. Among all genome-based inbreeding coefficients, the distribution of ${F}_{ROH}$ was similar to that of ${F}_{PED}$. The statistics and distribution of ${F}_{HBD}$ were similar to those of ${F}_{ROH\_30}$ and slightly higher than those of ${F}_{PED}$ and ${F}_{ROH}$.

**Table 1:** Summary statistics for the estimates of nine inbreeding coefficients

Inbreeding coefficient^a^	Min	Max	Mean	Median	SD^b^
${F}_{PED}$	0.000	0.412	0.093	0.081	0.058
${F}_{GRM}$	-0.197	0.403	0.032	0.013	0.095
${F}_{UNI}$	-0.095	0.401	0.060	0.050	0.072
${F}_{HOM}$	-0.159	0.385	0.031	0.010	0.095
${F}_{ROH}$	0.000	0.400	0.112	0.096	0.070
${F}_{ROH\_30}$	0.039	0.454	0.164	0.148	0.072
${F}_{ROH\_15}$	0.122	0.524	0.250	0.233	0.070
${F}_{HBD}$	0.040	0.437	0.162	0.147	0.068

^a^ ${F}_{PED}$, pedigree-based inbreeding coefficient; ${F}_{GRM}$, inbreeding coefficient based on genomic relationship matrix; ${F}_{UNI}$, inbreeding coefficient based on correlation between uniting gametes; ${F}_{HOM}$, inbreeding coefficient based on the observed vs expected number of homozygous genotypes; ${F}_{ROH}$, inbreeding coefficient based on ROH; ${F}_{ROH\_30}$, inbreeding coefficient based on ROH when minimum number of consecutive homozygous SNP included in the ROH was 30; ${F}_{ROH\_15}$, inbreeding coefficient based on ROH when minimum number of consecutive homozygous SNP included in the ROH was 15; ${F}_{HBD}$, inbreeding coefficient based on HBD

^b^ *SD* Standard deviation

**Fig. 3:**
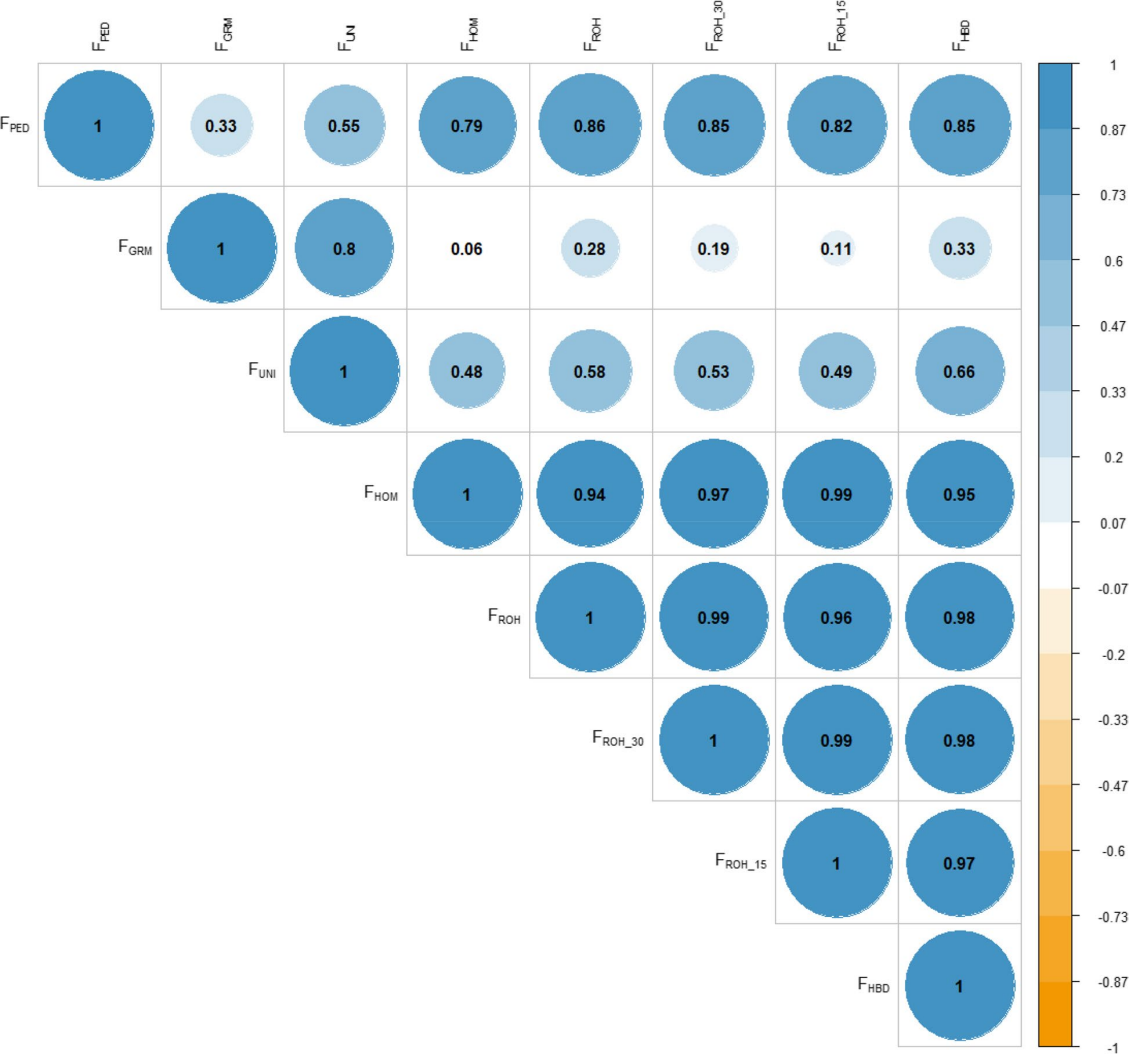
Pairwise Pearson correlations between different inbreeding coefficients. ${F}_{PED}$, pedigree-based inbreeding coefficient; ${F}_{GRM}$, inbreeding coefficient based on genomic relationship matrix; ${F}_{UNI}$, inbreeding coefficient based on correlation between uniting gametes; ${F}_{HOM}$, inbreeding coefficient based on the observed vs expected number of homozygous genotypes; ${F}_{ROH}$, inbreeding coefficient based on ROH; ${F}_{ROH\_30}$, inbreeding coefficient based on ROH when minimum number of consecutive homozygous SNP included in the ROH was 30; ${F}_{ROH\_15}$ inbreeding coefficient based on ROH when minimum number of consecutive homozygous SNP included in the ROH was 15; ${F}_{HBD}$, inbreeding coefficient based on HBD

**Fig. 4:**
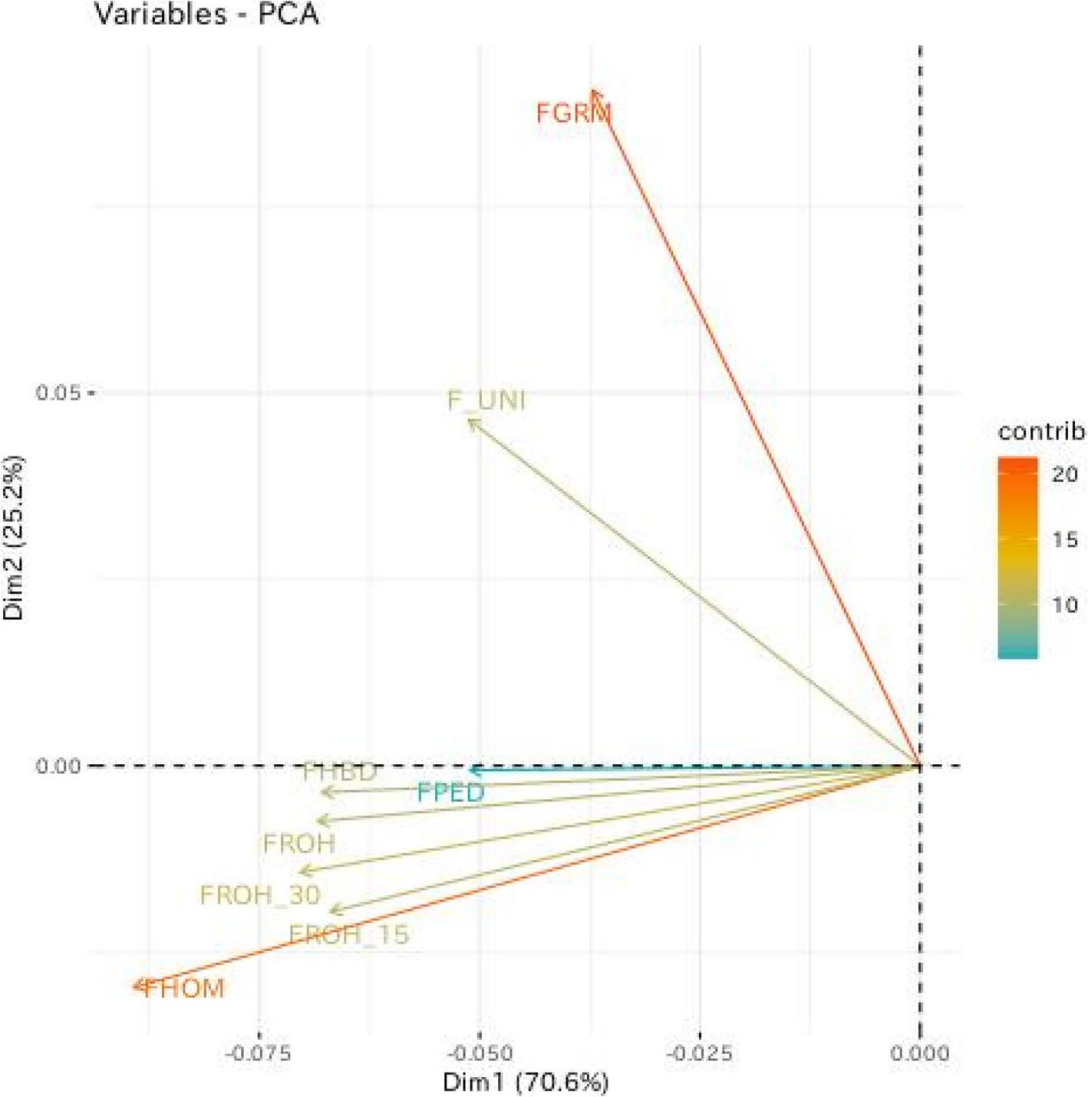
Scatterplot of the first two principal components (Dim1 and Dim2) in principal component analysis. Color scale indicates the contribution of each inbreeding coefficient on the first principal component. ${F}_{PED}$, pedigree-based inbreeding coefficient; ${F}_{GRM}$, inbreeding coefficient based on genomic relationship matrix; ${F}_{UNI}$, inbreeding coefficient based on correlation between uniting gametes; ${F}_{HOM}$, inbreeding coefficient based on the observed vs expected number of homozygous genotypes; ${F}_{ROH}$, inbreeding coefficient based on ROH; ${F}_{ROH\_30}$, inbreeding coefficient based on ROH when minimum number of consecutive homozygous SNP included in the ROH was 30; ${F}_{ROH\_15}$ inbreeding coefficient based on ROH when minimum number of consecutive homozygous SNP included in the ROH was 15; ${F}_{HBD}$, inbreeding coefficient based on HBD

The correlation coefficients of all estimated inbreeding coefficients are given in Fig. 3. Every pair of inbreeding coefficients was considered significant at *p* < 0.001. The correlations between ${F}_{PED}$ and two genome-based inbreeding coefficients ${F}_{GRM}$ and ${F}_{UNI}$ were weak, with range 0.33–0.55. The highest correlation with ${F}_{PED}$ was ${F}_{ROH}$ ($\rho =0.86$), followed by ${F}_{ROH\_30}$ and ${F}_{HBD}$ ($\rho =0.85$). Except for ${F}_{GRM}$ and ${F}_{UNI}$, there were strong correlations among genome-based inbreeding coefficients ($\rho \ge 0.94$). In particular, the correlations between ROH-based inbreeding coefficients and ${F}_{HBD}$ were around 1. The first two principal components (PCs) of the PCA captured more than 95% of the total variability of inbreeding coefficients (Fig. 4). The second PC distinguished ${F}_{GRM}$ and ${F}_{UNI}$ from the others, and grouped ${F}_{PED}$ and ${F}_{HBD}$ more closely.

**Fig. 5:**
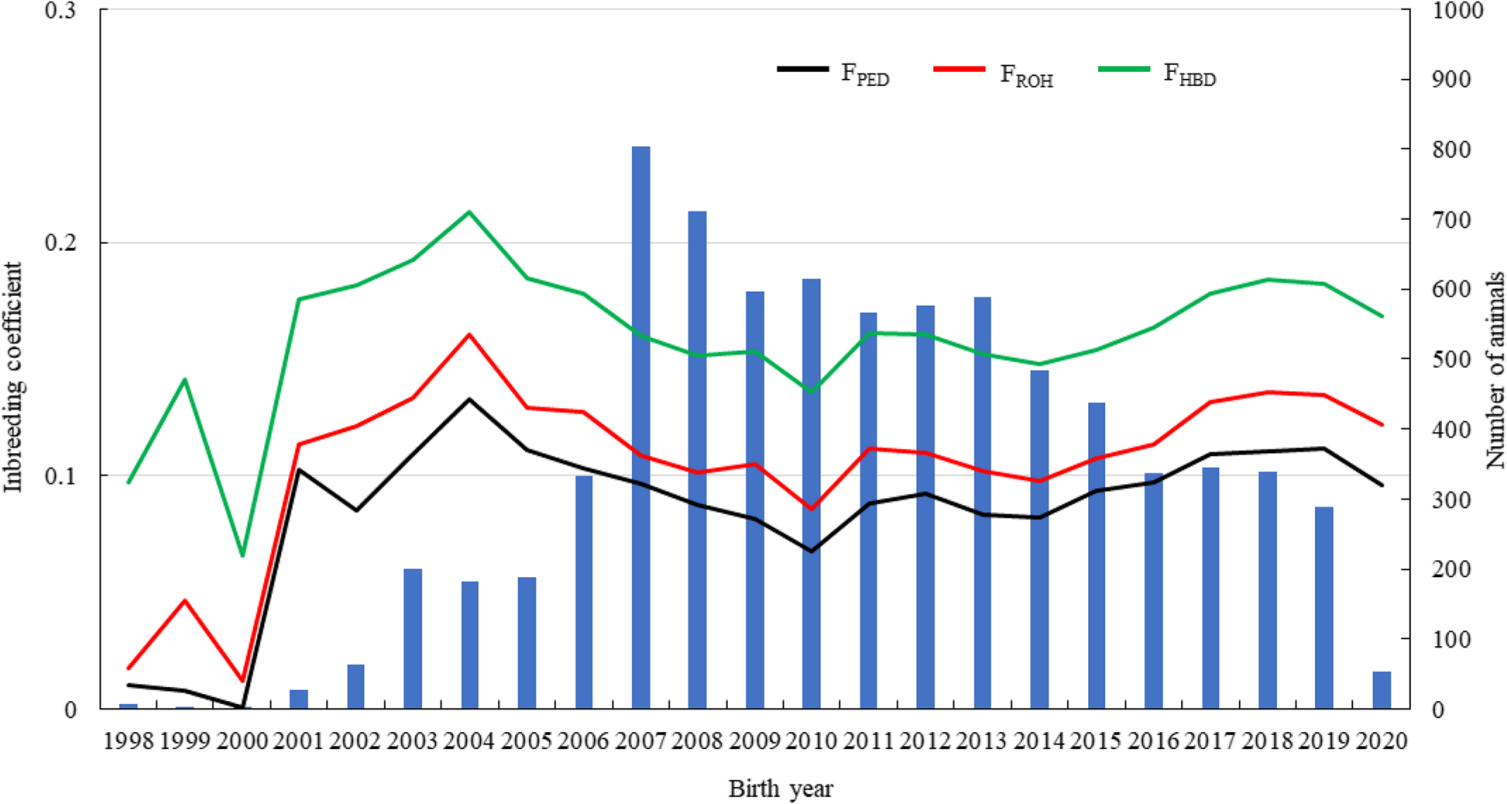
Trend lines of pedigree-based, ROH and HBD-based inbreeding coefficients (${F}_{PED}$, ${F}_{ROH}$ and ${F}_{HBD}$) and the number of animals at birth years from 1998 to 2020

The result of regression of the genome-based inbreeding coefficient on ${F}_{PED}$ (Table 2) showed a detailed comparison between pedigree- and genome-based inbreeding coefficients. The estimated regression coefficients of ${F}_{ROH}$, ${F}_{ROH\_30}$, ${F}_{ROH\_15}$ and ${F}_{HBD}$ were close to 1, with range 0.98–1.05. In particular, for ${F}_{HBD}$, the estimated regression coefficient was just 1, but the estimated intercept was larger than that for ${F}_{ROH}$. The ${F}_{ROH}$ and ${F}_{HBD}$ are parallel from the beginning, but ${F}_{PED}$ only slightly differs when no enough pedigree information seems to be (Fig. 5).

**Table 2:** Estimates of regression efficient and intercept (PSD^a^) of genome-based inbreeding coefficient on pedigree-based inbreeding coefficient

Estimate	Genome-based inbreeding coefficient^b^						
	${F}_{GRM}$	${F}_{UNI}$	${F}_{HOM}$	${F}_{ROH}$	${F}_{ROH\_30}$	${F}_{ROH\_15}$	${F}_{HBD}$
Regression coefficient	0.56 (0.03)	0.70 (0.02)	1.29 (0.02)	1.04 (0.01)	1.05 (0.01)	0.98 (0.01)	1.00 (0.01)
Intercept	-0.02 (0.003)	0.00 (0.002)	-0.09 (0.002)	0.02 (0.001)	0.07 (0.001)	0.16 (0.001)	0.07 (0.001)

^a^ *PSD* Posterior standard deviation

^b^ ${F}_{PED}$, pedigree-based inbreeding coefficient; ${F}_{GRM}$, inbreeding coefficient based on genomic relationship matrix; ${F}_{GRM0.5}$, inbreeding coefficient based on genomic relationship matrix with all marker frequencies of 0.5; ${F}_{UNI}$, inbreeding coefficient based on correlation between uniting gametes; ${F}_{HOM}$, inbreeding coefficient based on the observed vs expected number of homozygous genotypes; ${F}_{ROH}$, inbreeding coefficient based on ROH; ${F}_{ROH\_30}$, inbreeding coefficient based on ROH when minimum number of consecutive homozygous SNP included in the ROH was 30; ${F}_{ROH\_15}$, inbreeding coefficient based on ROH when minimum number of consecutive homozygous SNP included in the ROH was 15; ${F}_{HBD}$, inbreeding coefficient based on HBD

### Inbreeding depression

Table 3 presents the estimates of regression coefficients of inbreeding depression ($\widehat{\beta }$) for reproductive traits using different inbreeding coefficients. The $\widehat{\beta }$ for ${F}_{PED}$ was 2.1 for AFC, 0.63 for CD and -1.21 for GL, respectively; however, ${F}_{PED}$ were not significant for all traits. For CD, all $\widehat{\beta }$ for genome-based inbreeding coefficients were significant and, for GL, the $\widehat{\beta }$ for ${F}_{UNI}$ had a significant. For all traits, the values of $\widehat{\beta }/PSD$ for all genome-based inbreeding coefficients were larger than those for${F}_{PED}$.

**Table 3:** Estimates of the regression coefficients (PSD^a^) of inbreeding coefficients on reproductive traits

Inbreeding coefficient^c^	Trait^d^		
	AFC	CD	GL
${F}_{PED}$	2.1 (37.5)	0.63 (0.33)	-1.21 (1.89)
${F}_{GRM}$	15.4 (28.1)	0.58 (0.22)**	-2.13 (1.35)
${F}_{UNI}$	18.0 (29.9)	0.57 (0.25)*	-3.01 (1.51)*
${F}_{HOM}$	16.8 (28.5)	0.58 (0.22)**	-1.66 (1.36)
${F}_{ROH}$	16.9 (33.7)	0.74 (0.27)**	-2.07 (1.36)
${F}_{ROH\_30}$	11.5 (32.4)	0.62 (0.27)*	-2.01 (1.58)
${F}_{ROH\_15}$	24.4 (36.3)	0.64 (0.32)*	-1.90 (1.67)
${F}_{HBD}$	14.2 (33.0)	0.72 (0.26)**	-2.44 (1.54)

^a^ *PSD* Posterior standard deviation

^b^ *SD* Standard deviation

^c^ ${F}_{PED}$, pedigree-based inbreeding coefficient; ${F}_{GRM}$, inbreeding coefficient based on genomic relationship matrix; ${F}_{GRM0.5}$, inbreeding coefficient based on genomic relationship matrix with all marker frequencies of 0.5; ${F}_{UNI}$, inbreeding coefficient based on correlation between uniting gametes; ${F}_{HOM}$, inbreeding coefficient based on the observed vs expected number of homozygous genotypes; ${F}_{ROH}$, inbreeding coefficient based on ROH; ${F}_{ROH\_30}$, inbreeding coefficient based on ROH when minimum number of consecutive homozygous SNP included in the ROH was 30; ${F}_{ROH\_15}$, inbreeding coefficient based on ROH when minimum number of consecutive homozygous SNP included in the ROH was 15; ${F}_{HBD}$, inbreeding coefficient based on HBD

^d^ AFC, age at first calving; CD, calving difficulty; GL, gestation length

^*^ Significantly different from 0 at *p* < 0.05

^**^ Significantly different from 0 at *p* < 0.01

**Fig. 6:**
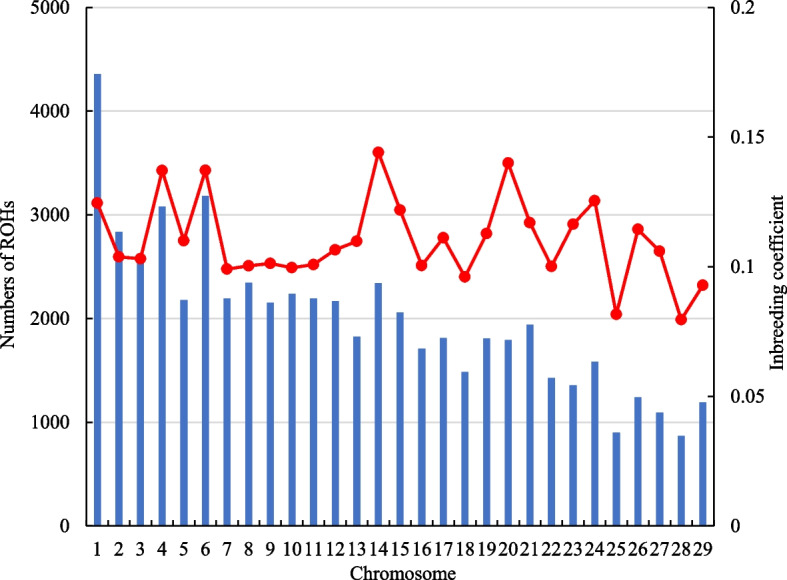
Number of ROH segments (blue vars) and average ROH-based inbreeding coefficient across the autosomal chromosomes (red line and markers)

The number of ROH segments varied across the chromosomes and contributed to the chromosomal ${F}_{ROH}$ (Fig. 6). The chromosomal ${F}_{ROH}$ was high in chromosomes 14 and 20, and low in chromosomes 25 and 28. Table 4 shows the only significant regression coefficients of inbreeding depressions per each chromosome using ${F}_{ROH}$ and ${F}_{HBD}$. For AFC, although there were no significant effects when using overall genome-level inbreeding coefficients, chromosomal ${F}_{ROH}$ provided significant negative effects in chromosomes 2 and 22, and positive effects in chromosomes 14 and 19. For CD, only chromosome 19 had a negative association whereas chromosomes 17 and 21 had positive associations. For ${F}_{HBD}$, there were no significances in chromosome 2 and 14 for AFC, and chromosome 21 for CD, but the differences in estimates between ${F}_{ROH}$ and ${F}_{HBD}$ were slight. For GL, the $\widehat{\beta }$ for both ${F}_{ROH}$ and ${F}_{HBD}$ provided significant positive effects in chromosome 5 (1.60 and 1.22), and negative effects in chromosome 26 (-1.22 and -1.70), respectively.

**Table 4:** Estimates of the regression coefficients (PSD^a^) of chromosomal inbreeding coefficients on reproductive traits

Trait^c^	Chromosome No	Chromosomal inbreeding coefficient^d^	
		${F}_{ROH}$	${F}_{HBD}$
AFC	2	-31.8 (14.3)*	-27.5 (19.2)
	14	21.8 (11.0)*	27.8 (16.6)
	19	26.0 (12.6)*	36.1 (18.1)*
	22	-29.9 (12.6)*	-35.2 (16.5)*
CD	17	0.24 (0.10)*	0.33 (0.15)*
	19	-0.18 (0.09)*	-0.33 (0.14)*
	21	0.20 (0.10)*	0.23 (0.13)
GL	5	1.60 (0.66)*	2.14 (0.82)**
	26	-1.22 (0.53)*	-1.70 (0.75)*

^a^ *PSD* Posterior standard deviation

^b^ *SD* Standard deviation

^c^ *AFC* Age at first calving, *CD* Calving difficulty, *GL* Gestation length

^d^ ${F}_{ROH}$, inbreeding coefficient based on ROH; ${F}_{HBD}$, inbreeding coefficient based on HBD

^*^ Significantly different from 0 at *p* < 0.05

^**^ Significantly different from 0 at *p* < 0.01

## Discussion

### Distribution of inbreeding coefficient

Inbreeding coefficient has been defined as a correlation [41] or a probability [50] and thus its range is is [-1, 1] or [0, 1], respectively. In this study, ${F}_{PED}$ was fitted to the latter definition and its mean was 0.093 using pedigree information of which maximum depth was 17. The accuracy of pedigree inbreeding estimates are largely dependent on the completeness and depth of the pedigree records [51, 52]. The shallow pedigree depth might underestimate the degree of inbreeding. Unlike${F}_{PED}$, there are various ways to estimate genome-based inbreeding coefficients, which provide different accepted spaces. The general spaces of genome-based inbreeding coefficients in this study are summarized: [-1, 1] for ${F}_{HOM}$, [$-1$,$+\infty$] for${F}_{UNI}$, [$-\infty$,$+\infty$] for ${F}_{GRM}$ and [0, 1] for ${F}_{ROH}$ and${F}_{HBD}$. The estimates of ${F}_{UNI}$ and ${F}_{GRM}$ can provide coefficients within [-1, 1], making it difficult to interpret and compare with traditional${F}_{PED}$. The methodology depends on the allele frequencies and do not work properly if allele frequencies are not those in the founder population. The negative value means that they are less inbred than in a hypothetical reference population with the frequencies used. However, a value above 1 is unrealistic because this means that more variability has been lost than initially existed in the base population [53]. In this study, the estimates of${F}_{HOM}$, ${F}_{UNI}$ and ${F}_{GRM}$ provided negative values with means below that of${F}_{PED}$. These results were consistent with previous studies of pig [54] and dairy cattle [55]. In particular, the minimum value of ${F}_{GRM}$ was the smallest ($-0.197$) among all inbreeding coefficients. The distribution of ${F}_{ROH}$ was closest to that of ${F}_{PED}$ whereas the means of ${F}_{ROH\_30}$ and ${F}_{ROH\_15}$ were higher and their SDs were almost the same compared to${F}_{ROH}$. This might be caused by the large number of ROH segments. Although there were no differences in the number of long ROH segments (> 8 Mb) among all ROH-based inbreeding coefficients, many short ROH segments (< 4 Mb) were detected by ${F}_{ROH\_30}$ and${F}_{ROH\_15}$. These resulted in increases in total ROH length and inbreeding coefficients. Sumreddee et al. [56] varied the minimum length of ROH segments from 0.5 to 8 Mb and showed that the ROH-based inbreeding coefficients linearly increased with fewer short ROH segments. The distribution of ${F}_{HBD}$ was higher than other estimates (excluding ${F}_{ROH\_30}$ and${F}_{ROH\_15}$), which is the same results reported by Zhang et al. [56].

### Relationship among inbreeding coefficients

The correlations between ${F}_{PED}$ and genome-based inbreeding coefficients varied greatly, with range 0.33–0.86. The estimates of ${F}_{GRM}$ and ${F}_{UNI}$ were weakly correlated with ${F}_{PED}$, consistent with results for Holstein–Friesian dairy cows [55], Holstein and Jersey bulls [57] and four Italian pig breeds [58]. This weak correlation would be due to inappropriate allele frequencies used in our analysis. We used the allele frequencies in the animals genotyped in the first periods from 1998 to 2003. However, the base population in the pedigree information were born from 1939. Thus, there is a long period between the base population for ${F}_{PED}$ and the reference population mimicking the founder one for ${F}_{GRM}$ and ${F}_{UNI}$. In particular, correlations between ${F}_{GRM}$ and other inbreeding coefficients (except for ${F}_{UNI}$) were lower than those among other inbreeding coefficients. This was also reported by Mastrangelo et al. [59] and Schaler et al. [20]. There were high correlations (> 0.94) among ${F}_{HOM}$, ${F}_{ROH}$, ${F}_{ROH\_30}$, ${F}_{ROH\_15}$ and ${F}_{HBD}$. Dadousis et al. [55] reported that correlations between ${F}_{HOM}$ and ${F}_{ROH}$ were > 0.85. Zhang et al. [54] also showed that correlations among ${F}_{HOM}$, ${F}_{ROH}$ and ${F}_{HBD}$ were > 0.85. The correlations between ${F}_{PED}$ and genome-based inbreeding coefficients obtained in this study were higher than those reported in several previous studies [57, 59, 60]. The reason would be because there is a great range of inbreeding values in this study. In addition, individuals in the former generations have low values of ${F}_{PED}$ and also low values of genome-based inbreeding coefficients, and the same for animals for high values. It is reinforced when using parameters that need to account with the frequencies in the founder population, in which these correlations go down as the animals in the intermediate generations (those with the frequencies similar to those used for computations) are those that these methodologies identified as close to "founders". In addition, our PCA results classified the inbreeding coefficients into two groups: ${F}_{GRM}$ was grouped with ${F}_{UNI}$, and all remaining inbreeding coefficients were clustered together. The common point of the first group was sensitivity to allele frequency. The ${F}_{GRM}$ and ${F}_{UNI}$ rely on variances of genotypes within individuals and correlations between parental gametes. These better fit the definition of the inbreeding coefficient in terms of correlation as proposed by Wright [41]. A second group (excluding ${F}_{HBD}$) was based on the number of homozygous SNPs that give equal weights to all alleles and corresponds to the definition by Malécot [50], relying on the probability that two homologous alleles in an individual are IBD. Although ${F}_{HBD}$ uses allele frequencies to calculate HBD probabilities, homozygous genotypes that are in long HBD segments have the same weight irrespective of their allele frequencies. Thus, ${F}_{HBD}$ was closer to the properties of the second group. The ${F}_{ROH}$ and ${F}_{HBD}$ are based on the IBD concept, resulting that they correlated better than others with ${F}_{PED}$. There were no definite criteria to determine the most suitable genome-based inbreeding coefficient representing the actual inbreeding level of a population. Thus, the genome-based inbreeding coefficient having a relatively high association with both pedigree- and other genome-based inbreeding coefficients was considered a good estimator. In regression analysis, regression coefficients of ${F}_{ROH}$ and ${F}_{HBD}$ on ${F}_{PED}$ were close to 1 (1.04 and 1.00, respectively). In addition, trends of ${F}_{ROH}$ and ${F}_{HBD}$ were similar to that of ${F}_{PED}$. These results suggested that ${F}_{ROH}$ and ${F}_{HBD}$ had a distinct advantage in estimating inbreeding level.

### Inbreeding depression

We found no significant effects of ${F}_{PED}$ on all reproductive traits in Japanese Black cattle. Several previous studies also reported no significant inbreeding depression associated with AFC and GL in Japanese Black cattle [23] and with AFC in Hereford cattle [56]. Compared with ${F}_{PED}$, using genome-based inbreeding coefficients tended to provide the larger estimates of inbreeding depression without increasing PSD. Thus, inbreeding depression on reproductive traits with genome-based inbreeding coefficients had lower *p*-values than pedigree-based inbreeding coefficients. In particular, all genome-based inbreeding coefficients showed significant associations with CD, and ${F}_{UNI}$ had a significant effect on GL. In Holstein dairy cattle, Bjelland et al. [61] showed that a 1% increase in genome-based inbreeding coefficient had an adverse effect of 0.04 for CD. This estimate was larger than our results (range 0.0058–0.0074), calculated by converting scales of estimates from 1 SD to 1% of inbreeding coefficients. The difference in the effects of inbreeding depression between pedigree- and genome-based inbreeding coefficients was due to many factors, including errors in pedigree records and depth, expected or actual IBD and the number of records. Our results and previous studies in pig [50] and in dairy cattle [60, 62] showed that using a pedigree-based inbreeding coefficient might underestimate inbreeding depression on female fertility traits. For CD and GL, the effects of inbreeding depression with ${F}_{ROH\_30}$ and ${F}_{ROH\_15}$ were lower than that with ${F}_{ROH}$. Because the loose criterion of parameter $L$ contributed to overestimating ROH-based inbreeding coefficients, inbreeding depression would be finally underestimated. However, for ${F}_{ROH}$, setting too large values of $L$ leads to many animals having a ${F}_{ROH}=0$. In this situation, ${F}_{ROH}$ might not identify better the IBD than IBS.

In previous studies of Japanese Black cattle, Nagai et al. [27] and Onogi et al. [28] used only the heterozygosity rate, which was independent of allele frequencies, which might be inappropriate for the populations they used. Recently, Caballero et al. [63] compared several estimators of inbreeding coefficients and inbreeding depression in simulated data of an assumed Iberian pig population. They concluded that estimates of ${F}_{ROH}$ were very precise in most simulation scenarios whereas estimates from simple allele frequencies of homozygous marker (${F}_{HOM}$) could not be used to estimate inbreeding depression. In our analysis, there were no significant differences in estimates of inbreeding depression between ${F}_{ROH}$ and ${F}_{HBD}$. Therefore, ${F}_{ROH}$ or ${F}_{HBD}$ could provide precise estimates of inbreeding depression regardless of target population.

The inbreeding depressions for all chromosomes were estimated simultaneously for ${F}_{ROH}$ and ${F}_{HBD}$ because the correlations among ROH-based chromosomal inbreeding coefficients were weak (Fig. S1). This regression analysis showed several significant inbreeding depressions associated with chromosomal ${F}_{ROH}$ and ${F}_{HBD}$ for all reproductive traits. The correlation between the contribution of a chromosome to the genome-wide inbreeding and its effect on inbreeding depression of reproductive traits was not high, consistent with results for growth traits in Hereford cattle [56]. This implies that chromosomes with high inbreeding contributions carried no genes affecting the reproductive traits investigated in this study. In addition, the inbreeding coefficient of each chromosome was not proportional to chromosomal length (Fig. S2). This might be because recombination rate locally influences ROH patterns [64] or selection pressure shapes the ROH landscape [56]. Although genome-wide inbreeding coefficients had an adverse impact on AFC and CD, favorable effects of ${F}_{ROH}$ and ${F}_{HBD}$ were found in chromosomes 2 and 22 for AFC and in chromosome 19 for CD. For AFC, ${F}_{ROH}$ in chromosome 14 had an adverse effect and had become high during the last 10 years (Fig. S3). The information on chromosomal inbreeding depression and its trend could be beneficial because we could control specific chromosomal inbreeding coefficients with adverse or favorable effects on target traits by mating decision [65, 66] and thus suppress inbreeding depression.

For all inbreeding coefficients, Pearson’s correlations and Spearman's rank correlation coefficients between estimated breeding values by statistical models with inbreeding and without inbreeding were above 0.99. In our analysis, the correlations between several genome-based inbreeding coefficients and ${F}_{PED}$ were high, resulting that the effect including genomic-based inbreeding coefficients instead of ${F}_{PED}$ on improvement of genetic evaluation is limited. However, when the pedigree data is missing or contains errors, incomplete pedigree information would result in underestimating ${F}_{PED}$ and low correlation between ${F}_{PED}$ and genome-based inbreeding coefficients. In this situation, genome-based inbreeding coefficient might facilitate the genetic improvement.

### Further perspectives

This study used genotyped cows imputed from BovineHD genotypes of 651 bulls. Although few studies have investigated the effect of imputation on the genome-based inbreeding coefficient, Dadousis et al. [55] showed unreasonable homozygosity levels after imputation and hypothesized that imputation might cause extreme genomic inbreeding values. In our analysis, the ratios of short ROH segments were quite high for ${F}_{ROH\_30}$ and ${F}_{ROH\_15}$, possibly caused by using imputed SNP data. Because the imputed SNP data were related to many confounding factors such as the quality of the sample data and the properties of the phased reference panel, further research should identify all possible factors that influence genome-based inbreeding coefficients.

Our analysis focused on inbreeding depression at the overall genome and chromosomal levels. When segment-based, inbreeding depression could be detected at the chromosomal region level. Several recent studies explored genome-wide ROH patterns and inbreeding depression in cattle populations using BovineSNP50 arrays [61, 67, 68]. However, Ferencakovic et al. [69] stated that the BovineSNP50 array might underestimate the number of fragments of length 1–4 Mb. Zhao et al. [70] showed the power of high-density SNP arrays (503,579 SNPs) for identification of small ROH associated with body weight, calving ease and stillbirth in Chinese Wagyu beef cattle. Therefore, further study using high-density SNP arrays for detection of inbreeding depression at the chromosomal region level is required.

## Conclusions

This study provided a comparative analysis of nine inbreeding measures, pedigree- and genome-based, and quantified the potential inbreeding depression on the reproductive traits in Japanese Black cattle. The ROH- and HBD-based inbreeding coefficients had relatively high associations with both pedigree- and other genome-based inbreeding coefficients, and thus could be considered good estimators for qualifying inbreeding level. Genomic inbreeding measures seemed to capture more phenotypic differences than pedigree-based measures. As a point of caution, the ROH-based measure required appropriate parameter setting because the loose criterion for detecting ROH segments could overestimate inbreeding level and underestimate inbreeding depression. Moreover, we found several significant effects of inbreeding coefficients per chromosome on all reproductive traits using ROH- and HBD-based measures. We could suppress inbreeding depression or facilitate the genetic improvement by controlling specific chromosomal inbreeding coefficients with adverse or favorable effects on target traits. Therefore, information on chromosomal inbreeding depression could be beneficial for an animal breeding program.

## Supplementary Information

**Additional file 1: Figure S1.** Pairwise Pearson correlations of ROH-based chromosomal inbreeding coefficients among autosomal chromosomes

**Additional file 2: Figure S2.** Scatter plot of ROH-based chromosomal inbreeding coefficients

**Additional file 3: Figure S3.** Trend lines of ROH-based inbreeding coefficients in chromosomes 2, 14, 19 and 22 which were associated with age at first calving at birth years from 1998 to 2020

## Data Availability

The datasets analyzed during the present study are not available because it is property of the Japanese Black cattle producers in Japan and this information is commercially very sensitive. A request to the data from this study may be sent to the corresponding author, Motohide Nishio (mtnishio@affrc.go.jp).
