# Supplementary material for: Comparing pedigree and genomic inbreeding coefficients, and inbreeding depression of reproductive traits in Japanese Black cattle
Source: BMC Genomics. 2023 Jul 5;24:376. doi: 10.1186/s12864-023-09480-5 (PMC10321020; doi:10.1186/s12864-023-09480-5)
Supplement: Supplementary file 1 — Additional file 1: Figure S1. Pairwise Pearson correlations of ROH-based chromosomal inbreeding coefficients among autosomal chromosomes [file 12864_2023_9480_MOESM1_ESM.pdf]

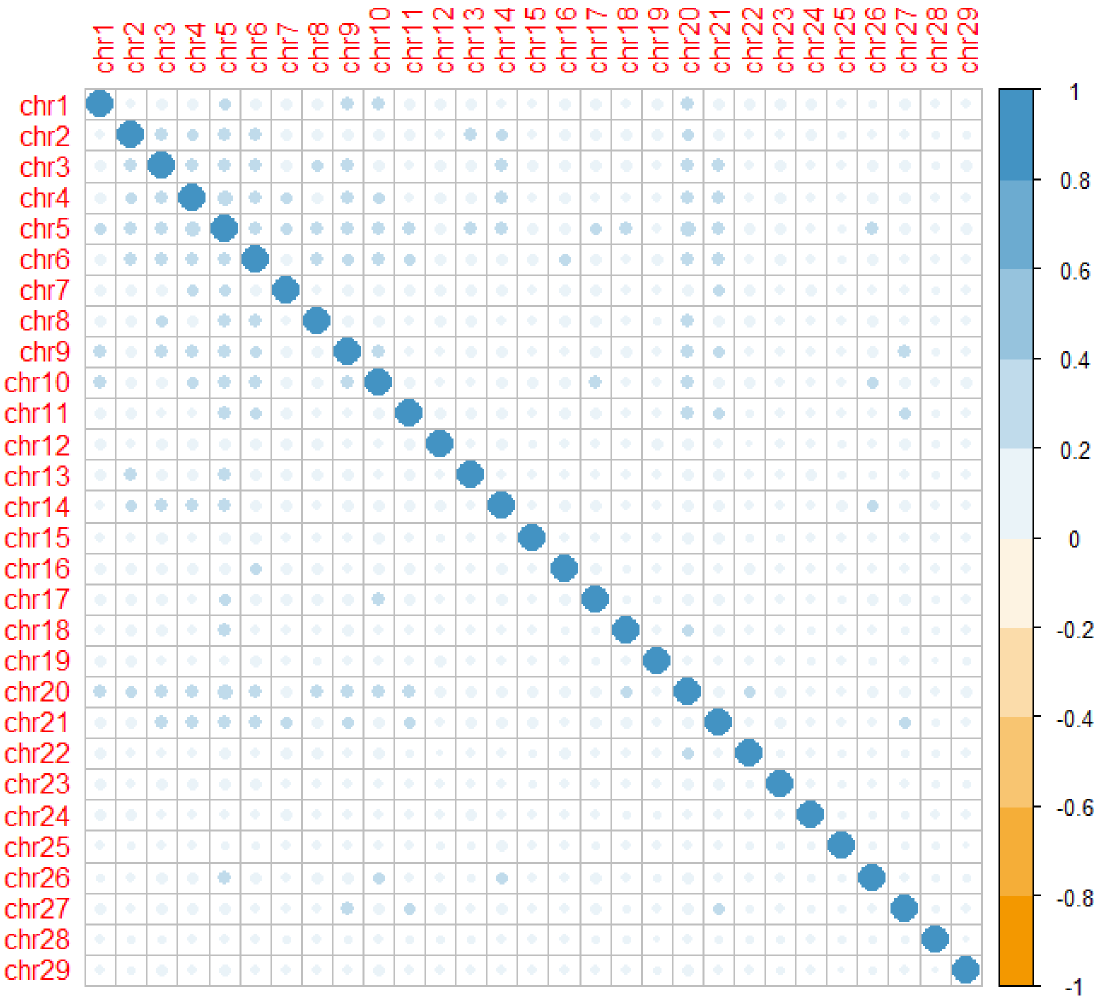

Additional file 1: Figure S1. Pairwise Pearson correlations of ROH-based chromosomal inbreeding coefficients among autosomal chromosomes
