# Supplementary figures and images for: Comparing pedigree and genomic inbreeding coefficients, and inbreeding depression of reproductive traits in Japanese Black cattle
Source: BMC Genomics. 2023 Jul 5;24:376. doi: 10.1186/s12864-023-09480-5 (PMC10321020; doi:10.1186/s12864-023-09480-5)

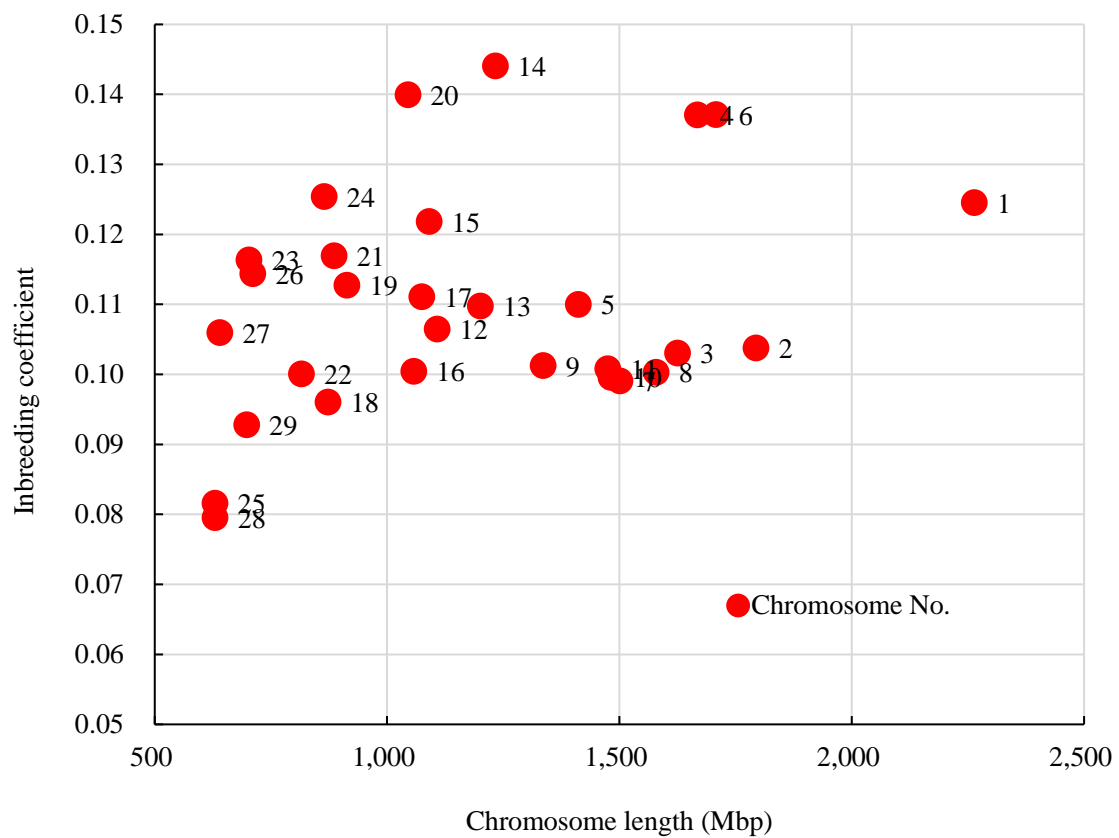

**Additional file 3: Figure S2.** Scatter plot of ROH-based chromosomal inbreeding coefficients

Supplement: Supplementary file 2 — Additional file 2: Figure S2. Scatter plot of ROH-based chromosomal inbreeding coefficients [file 12864_2023_9480_MOESM2_ESM.pdf]
