# Supplementary material for: Comparing pedigree and genomic inbreeding coefficients, and inbreeding depression of reproductive traits in Japanese Black cattle
Source: BMC Genomics. 2023 Jul 5;24:376. doi: 10.1186/s12864-023-09480-5 (PMC10321020; doi:10.1186/s12864-023-09480-5)
Supplement: Supplementary file 3 — Additional file 3: Figure S3. Trend lines of ROH-based inbreeding coefficients in chromosomes 2, 14, 19 and 22 which were associated with age at first calving at birth years from 1998 to 2020 [file 12864_2023_9480_MOESM3_ESM.pdf]

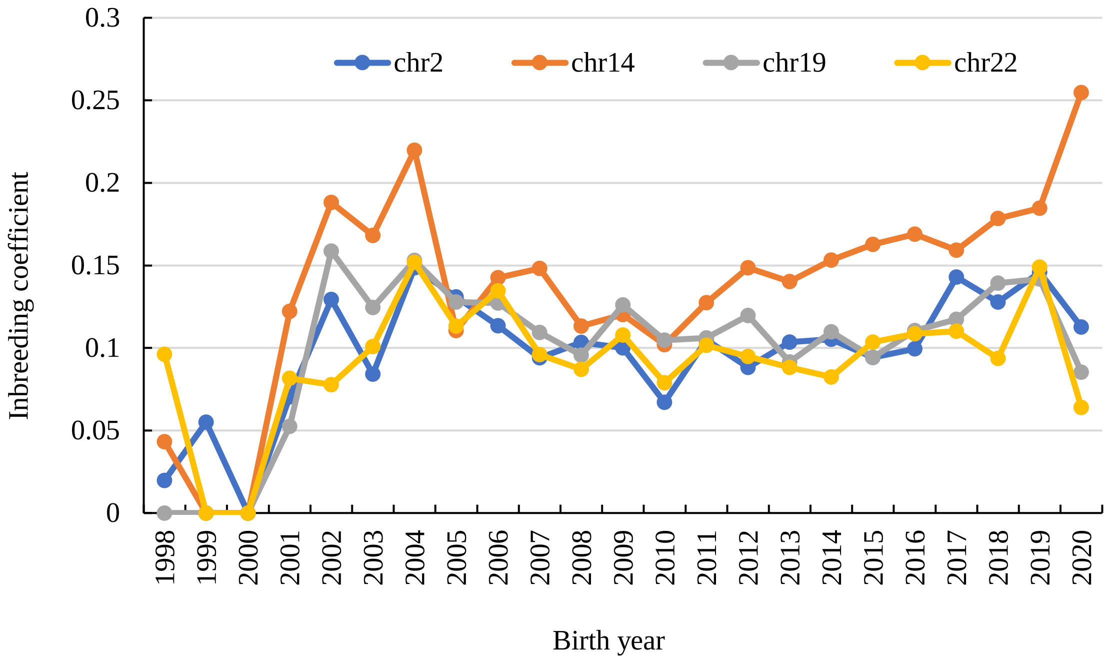

Additional file 4: Figure S3. Trend lines of ROH-based inbreeding coefficients in chromosomes 2, 14, 19 and 22 which were associated with age at first calving at birth years from 1998 to 2020
